# Acupuncture with manual and electrical stimulation for labour pain: a longitudinal randomised controlled trial

**DOI:** 10.1186/1472-6882-14-187

**Published:** 2014-06-09

**Authors:** Linda Vixner, Erica Schytt, Elisabet Stener-Victorin, Ulla Waldenström, Hans Pettersson, Lena B Mårtensson

**Affiliations:** 1 Department of Women’s and Children’s Health, Division of Reproductive Health, Karolinska Institutet, Retzius väg 13A, 171 77 Stockholm, Sweden; 2 School of Health and Social Studies, Dalarna University, 791 88 Falun, Sweden; 3 Centre for Clinical Research Dalarna, Nissers väg 3, 791 82 Falun, Sweden; 4 Department of Physiology, Sahlgrenska Academy, Institute of Neuroscience and Physiology, University of Gothenburg, 405 30 Gothenburg, Sweden; 5 Department of Clinical Science and Education, Södersjukhuset Karolinska Institutet, Stockholm, Sweden; 6 School of Health and Education, University of Skövde, P.O. Box 408, 541 28 Skövde, Sweden

## Abstract

**Background:**

Acupuncture is commonly used to reduce pain during labour despite contradictory results. The aim of this study is to evaluate the effectiveness of acupuncture with manual stimulation and acupuncture with combined manual and electrical stimulation (electro-acupuncture) compared with standard care in reducing labour pain. Our hypothesis was that both acupuncture stimulation techniques were more effective than standard care, and that electro-acupuncture was most effective.

**Methods:**

A longitudinal randomised controlled trial. The recruitment of participants took place at the admission to the labour ward between November 2008 and October 2011 at two Swedish hospitals . 303 nulliparous women with normal pregnancies were randomised to: 40 minutes of manual acupuncture (MA), electro-acupuncture (EA), or standard care without acupuncture (SC). Primary outcome: labour pain, assessed by Visual Analogue Scale (VAS). Secondary outcomes: relaxation, use of obstetric pain relief during labour and post-partum assessments of labour pain. The sample size calculation was based on the primary outcome and a difference of 15 mm on VAS was regarded as clinically relevant, this gave 101 in each group, including a total of 303 women.

**Results:**

Mean estimated pain scores on VAS (SC: 69.0, MA: 66.4 and EA: 68.5), adjusted for: treatment, age, education, and time from baseline, with no interactions did not differ between the groups (SC vs MA: mean difference 2.6, 95% confidence interval [CI] -1.7-6.9 and SC vs EA: mean difference 0.6 [95% CI] -3.6-4.8). Fewer number of women in the EA group used epidural analgesia (46%) than women in the MA group (61%) and SC group (70%) (EA vs SC: odds ratio [OR] 0.35; [95% CI] 0.19-0.67).

**Conclusions:**

Acupuncture does not reduce women’s experience of labour pain, neither with manual stimulation nor with combined manual and electrical stimulation. However, fewer women in the EA group used epidural analgesia thus indicating that the effect of acupuncture with electrical stimulation may be underestimated. These findings were obtained in a context with free access to other forms of pain relief.

**Trial registration:**

ClinicalTrials.gov: NCT01197950.

## Background

Acupuncture is commonly used to reduce pain during labour [1] despite contradictory results from studies evaluating its effectiveness [2]. In two studies, acupuncture has been reported to reduce pain [3,4], whereas other studies have found no evidence of pain reduction compared to sham control or standard care [5-7], although it has been found that those receiving acupuncture treatment had a lower frequency of epidural analgesia and/or pethidine use [5,8]. Furthermore, our own research group has demonstrated that sterile water injections, which give high intensity sensory stimulation, reduced the intensity of labour pain more effectively than acupuncture using manual stimulation of the needles [9].

Acupuncture involves puncturing the skin with thin sterile needles at defined acupuncture points. After placement, the needles are stimulated either manually or electrically. In acupuncture with manual stimulation (manual acupuncture; MA), the needles are twisted back and forth until a sensation of DeQi is achieved, which is described as a feeling of numbness, soreness or heaviness. In acupuncture with electrical stimulation (electro-acupuncture; EA), the needles are connected to a stimulator delivering either high or low frequency impulses, or a combination of both. The “dose” of acupuncture is determined by different factors such as number of needles, depth of insertion, type and intensity of stimulation; for MA by the number of times the needles are manipulated until DeQi is achieved, and for EA by the frequency and intensity of the electrical stimulation. In addition, the effect of acupuncture treatment is associated with the patient’s expectations and perception of the stimulation [10].

Whether the dose and mode of acupuncture stimulation was given in an optimal way in previous studies could be discussed, and if acupuncture treatment is more effective at certain stages of labour remains unknown. Pain relief through MA and EA have many similarities but there are also a number of differences, both regarding the type of afferents activated (EA: Aβ/δ-types, MA: all types, particularly C-types) and which opioid peptides are released [11]. As a consequence, one could expect that a combination of manual and electrical stimulation would lead to more effective pain relief than manual stimulation alone [11], but this has not yet been evaluated in the context of labour pain.

The aim of the present study was to evaluate the effectiveness of acupuncture with manual stimulation of the needles as well as the combination of manual and electrical stimulation in reducing labour pain, compared with standard care without any form of acupuncture. Our hypothesis was that the combination of manual and electrical stimulation would be the most effective stimulation.

The primary outcome of the study was women’s self-assessment of labour pain. Secondary outcomes were: experience of relaxation, use of epidural analgesia, and satisfaction with pain relief. We also monitored the following outcomes: mode of delivery, other forms of pain relief, augmentation of labour, duration of labour, perineal trauma, newborn Apgar score, umbilical cord pH and Base Excess, and transfer of the infant to neonatal clinic. Finally, we studied possible negative side-effects of the acupuncture treatment.

## Methods

The study was designed as a longitudinal randomised controlled trial. A full description of the study design has been presented previously [12]. The study protocol followed CONSORT [13] and STRICTA [14] recommendations and the rationale of acupuncture was based on Western medical theories [11,15,16].

The recruitment of participants took place between November 2008 and October 2011 at two hospital delivery wards in Sweden. Inclusion criteria for participation were: healthy nulliparous women with normal singleton pregnancies and a foetus in cephalic presentation admitted to the delivery ward in an active or latent phase of labour, after a spontaneous onset of labour. Women were excluded if they had received any pharmacological pain relief within the 24 hours prior to inclusion into the study with the exception of paracetamol, or if they were given oxytocin at the time point of allocation. After oral and written consent were obtained, the women were randomly allocated into one of three groups: manual acupuncture (MA), electro-acupuncture (EA), or standard care (SC). The randomisation was computerised by the first author LV and generated a list of codes from 1 to 303, with each code linked to one of the three groups. The randomisation was conducted in blocks with the length of 9, 12 and 15 which varied randomly. The participating midwives had varied training and experience of administering acupuncture treatment. A one-day study-specific course was given before study start, which included theory and hands-on-training in MA and EA.

### Intervention

All women in the trial received care from midwives throughout labour and birth, and from obstetricians in cases of deviation from normal progress, according to Swedish clinical practice. The participants in all three groups had access to all pharmacological and non-pharmacological analgesia available in Swedish maternity care. However, women in the SC group did not have access to any form of acupuncture. After randomisation and when requesting pain relief, women in the MA and EA groups were treated with 13–21 needles, at 3 bilateral distal points and 4–8 bilateral local points, all within the same somatic area as the cervix and uterus. The local points were chosen with regard to the pain location. The needles were removed after 40 minutes [12]. In the MA group, the needles were inserted and stimulated manually until DeQi was achieved and thereafter stimulated at ten-minute intervals. In the EA group, the needles were inserted and first stimulated manually until DeQi was achieved, then eight of the local needles were connected to an electrical stimulator which was set at a high frequency (80 Hz) stimulation. The decision regarding which local needles were to be connected to the stimulator was made by the midwife. The woman adjusted the intensity of the electrical stimulation herself to a level just under the pain threshold. The remaining needles were stimulated manually every ten minutes by the midwife until DeQi was achieved. In this way, women in the EA group received a combination of electrical and manual stimulation. MA or EA treatment was repeated after two hours, and thereafter made available on request. After the first treatment with acupuncture, women in the MA end EA groups had access to all the other pharmacological and non-pharmacological methods of pain relief available on the delivery wards. Women in the SC group had access to all forms of pain relief with the exception of acupuncture, and the choice of which pain relief that was used was made by the woman and the midwife together.

### Outcome variables

Primary outcome: The women assessed their labour pain using a Visual Analogue Scale (VAS), which has a 100 mm horizontal ungraded line with two endpoints: ‘no pain’ and ‘worst imaginable pain’. These assessments were made before the first treatment, immediately after the first treatment, every 30 minutes for five hours, and thereafter every hour until birth, or until epidural analgesia was administered. A different person from the one who administered the intervention (help nurse or midwife) assisted the women in the procedure of measuring pain and relaxation, however, blinding was not possible.

Secondary outcomes: Relaxation was also assessed using a VAS, the endpoints being: ‘relaxed’ and ‘very tense’. Use of obstetric pain relief and post-partum assessments of labour pain were also registered. A complete description of the outcome variables has been published previously [12].

### Statistical analyses

The sample size calculation was based on the primary outcome: women’s assessment of labour pain. A difference of 15 mm on VAS [17] was regarded as clinically relevant, and the detection of such a difference would require 41 women per group. A previous study [9] reported that only 47% of the women had registered data on pain or relaxation two hours after the first treatment (personal communication with Dr Mårtensson, January 2008), and compensation for a similar dropout rate would require 88 women per group. Finally, we compensated for an additional dropout rate of 15% due to women discontinuing their participation in the study or midwives’ being unable to participate because of a heavy workload. In total, we aimed to include 303 women, i.e. 101 women per group. The Bonferroni adjusted significant level was 0.017, power 0.80, and a standard deviation of 20.4 mm was based on historical data [7].

**Figure 1:**
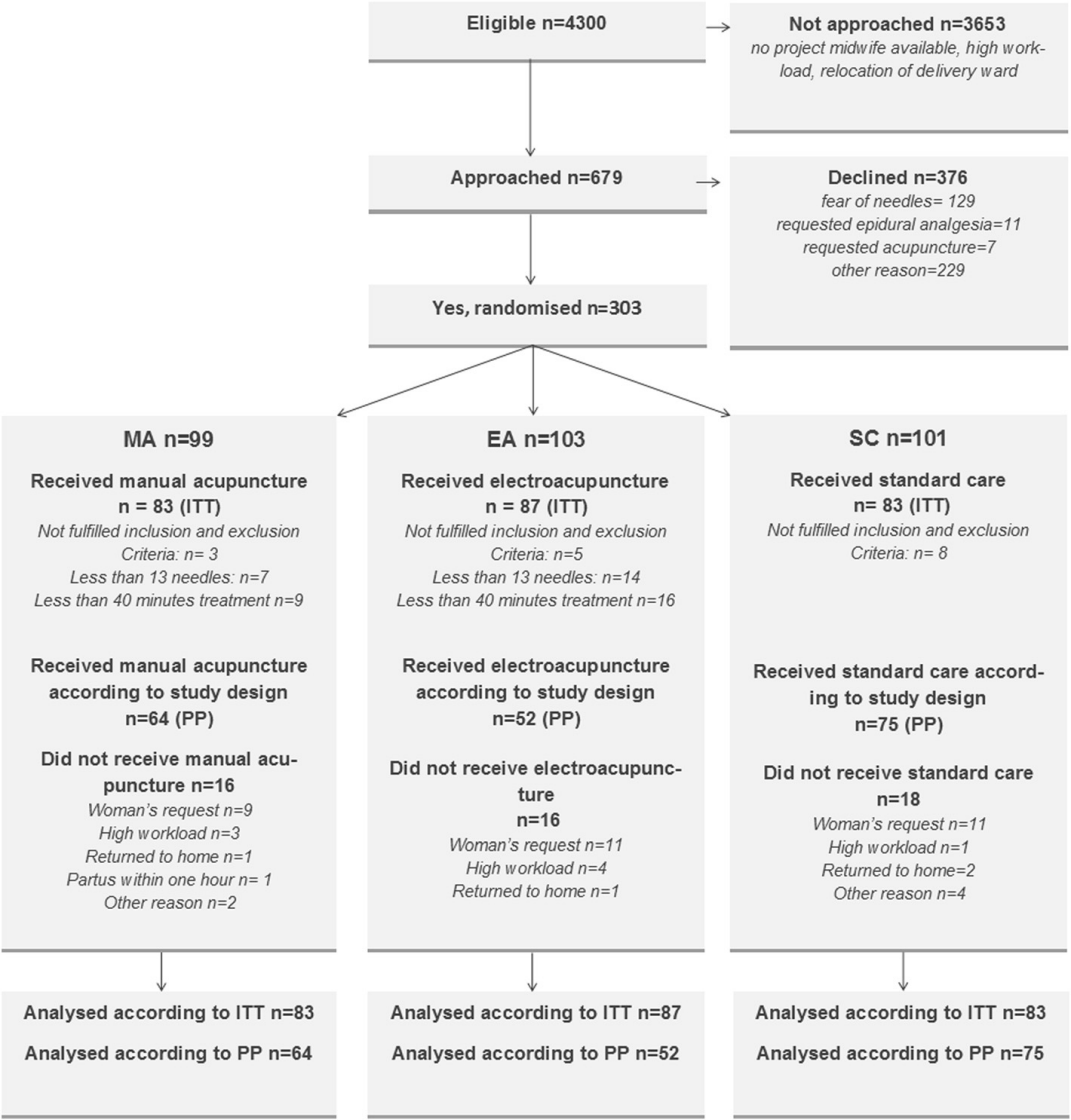
**Flow chart of the study participants.** MA = Manual acupuncture, EA = Electro-acupuncture, SC = Standard Care, ITT = Intention to treat, PP = Per protocol.

Analyses of the primary outcome were according to intention to treat (ITT) *and* per protocol (PP). The ITT analysis included all women randomised whereas the PP analysis excluded women who were randomised despite them not fulfilling inclusion criteria (MA n = 3; EA n = 5; SC n = 8), or who did not receive the interventions as planned (MA n = 16; EA n = 30; see Figure 1). All results presented in this article are analysed according to ITT.

Baseline characteristics are reported as means for continuous variables and percentages for discrete variables (Table 1). For the primary outcome of labour pain, a linear mixed model for repeated measures was performed to investigate associations between treatment (MA, EA, SC) and pain scores on VAS over time. According to protocol, pain assessments should have been made every 30 minutes but for practical reasons such as contractions and toilet visits, pain scores were assessed at slightly varying time points. For this reason they were categorised into time intervals and the mean pain score within each 30 minute interval was calculated. Firstly, a fixed effect model was estimated with all main effects (treatment, time) and adjusted for the background factors that differed between groups despite the randomisation. Secondly, an interaction was added between time and treatment to study if the three groups differed at different time points. For both models it was assumed that covariance structure for time was a first order Autoregressive Model AR(1). Since the estimated VAS results in these two models were very similar, the model was primarily used without interaction, which estimated fewer parameters (n = 23) than the model with interaction (n = 53).

**Table 1:** Characteristics of women allocated to manual acupuncture (MA), electro-acupuncture (EA), and standard care (SC), and their infants

	**Randomised**			**Analysed**		
**Characteristics**	**MA (n** = **99)**	**EA (n** = **103)**	**SC (n** = **101)**	**MA (n** = **83)**	**EA (n** = **87)**	**SC (n** = **83)**
*Socio-demographic background*						
Age (years), mean (SD)	26.5 (4.7)	27.7 (4.6)	28.1 (5.1)	26.5 (4.8)	27.6 (4.6)	28.3 (5.0)
Born in Sweden (%)	91.3	89.8	90.7	91.3	89.7	90.2
Higher education (%)	35.0	45.5	53.5	35	44.8	54.2
Solo parent (%)	15.2	19.4	15.8	14.5	18.4	15.7
Smoking 3 months prior to admission to antenatal clinic (%)	26.7	23.1	20.5	23.0	19.5	19.7
Body mass index in early pregnancy, mean (SD)	24.7 (4.8)	24.2 (3.9)	25.1 (4.1)	24.4 (5.0)	24.2 (3.8)	24.9 (4.1)
*Previous acupuncture experience*						
Yes, for pain (%)	17.1	23.8	14.5	17.1	24.1	13.7
Yes, for other than pain (%)	17.1	15.3	13.3	17.1	15.5	12.5
*Status at admission to labour ward*						
Gestational week, mean	40 + 0	40 + 0	40 + 0	40 + 0	39 + 6	40 + 0
Undiagnosed breach presentation (%)	0	0	1	0	0	1.2
Cervix dilatation (cm), mean (SD)	3.6 (1.6)	4.0 (1.7)	3.6 (1.7)	3.6 (1.5)	4 (1.6)	3.6 (1.8)
Rupture of membranes (%)	30.1	28.4	33.3	30.5	28.7	33.3
Pharmacological pain relief prior to recruitment, except for paracetamol (%)	4.0	2.9	11.9	2.4	3.4	8.4
Treatment with oxytocin at the time point of allocation (%)	1.0	1.9	1.0	1.2	2.3	0
*Infant*						
Head circumference (cm), mean (SD)	35.0 (1.4)	35.0 (1.3)	35.0 (1.3)	34.9 (1.4)	34.9 (1.3)	35 (1.3)
Birth weight (grams), mean (SD)	3509 (408)	3593 (451)	3656 (488)	3508 (410)	3590 (456)	3654 (493)

MA = Manual acupuncture. EA = Electro-acupuncture. SC = Standard care, SD = Standard deviation.

For the secondary outcomes, appropriate methods were selected for the measurement level of the outcome variables. Relaxation levels measured by VAS were analysed in the same way as the pain scores. To estimate the time from baseline to delivery, as well as the time from baseline to epidural analgesia, we used a Kaplan-Meier survival curve and a Cox regression model to make adjustments for the differences between the treatment groups at baseline (age, education). For discrete variables, logistic regression analyses were used, and adjustments were made for differences in background factors, using SC as the reference. These outcomes were: pain assessed as ‘worse than expected’ (much worse than expected + worse than expected *vs.* as expected + milder than expected + much milder than expected), use of pain relief other than acupuncture, ‘sufficient’ pain relief (enough *vs.* not enough), overall assessment of acupuncture for reducing pain and increasing relaxation (very effective + rather effective *vs.* not very effective + not effective at all), if the woman would choose the same treatment in future childbirth (yes *vs.* no), satisfaction with the allocation (yes *vs.* no), mode of delivery, estimated blood loss, augmentation of labour, perineal trauma, infant outcomes, perception of the midwife (positive *vs.* negative), support from the midwife (yes, to a high extent *vs* yes, to a rather high extent + no, to a rather low extent + no, not at all), number of acupuncture treatments, treatment with fewer than13 needles or less than 40 minutes of treatment, midwife’s acupuncture skills (very competent + quite competent *vs.* not very competent + not competent at all) and negative side effects of the acupuncture treatment (yes *vs.* no). For the following continuous variables we used a two way Anova analysis: cervix dilatation when epidural analgesia was administrated, umbilical cord pH, number of needles, and duration of acupuncture treatment. P-values less than 0.05 were regarded as statistically significant. Analyses were conducted using IBM SPSS STATISTICS 21.0, for Windows.

### Registration

This randomised controlled trial (RCT) was registered at ClinicalTrials.gov: NCT01197950. Due to a misunderstanding in the process, the study was not registered in Clinical trials before commencing the data collection. The trial was registered one year prior completion of the study (August 26, 2010). No changes were made from inclusion of the first woman until completion of the study. The authors confirm that all ongoing and related trials for these interventions are registered.

### Ethics statement

The study has no foreseeable risks but may cause minor discomfort in the form of tiredness or minor bruising. The women were informed that 1) participation in the study was voluntary, 2) their decision whether or not to participate would not affect their current or future treatment, 3) if they decided to participate they were free to withdraw at any time and 4) all questionnaires and blood samples would be unidentified. The women who agreed to participate in the study signed a consent form. The study was approved by the Regional Ethical Review Board, University of Gothenburg, 2008-05-15, Dnr: 136–08.

## Results

Recruitment and participation are presented in the flow-chart (Figure 1). Approximately 4300 women were eligible, 679 were informed and asked to participate in the study, and 303 consented to participate. Of the 303 women randomised, data on the primary outcome were obtained from 253 women: 83 in the MA group, 87 in the EA group, and 83 in the SC group.

Characteristics of the women randomised and those included in the final analyses are presented in Table 1. No differences were found between the three groups, with the exception of women in the SC group being older and more educated than women in the MA group. Consequently, the subsequent analyses were adjusted for these two variables. To test the representativity of our sample we obtained data on maternal age, relationship status, smoker or non-smoker, and body mass index for all women who were eligible for the study. Our study sample did not differ from this larger group except regarding smoking which was less common in the study sample.

The mean time (minutes) from inclusion in the study until the first treatment occasion did not differ between the groups; MA: 19.8 (SD 53.9), EA: 15.6 (SD 24.8), and SC: 30.2 (SD 74.4). The received intervention in terms of local and distal acupuncture points is presented in Table 2. The mean duration of the first acupuncture treatment was over 40 minutes in both treatment groups; 50 (SD 10.3) minutes in the MA group and 48 (SD 12.4) minutes in the in the EA group (*p* = 0.06). The mean number of needles was more than 13 in both groups: MA 14.9 (SD 2.8) and EA 14.9 (SD 3.2). Few women received a second treatment; MA 10.8% (n = 9), EA 8% (n = 7), and only one woman, who was in the EA group, received a third treatment. There were no statistically significant differences between the groups regarding the number of women who did not receive the first acupuncture treatment as intended (less than 13 needles (*p* = 0.14) or less than 40 minutes (*p* = 0.29)) (Figure 1).

**Table 2:** Acupuncture points (%)

	**MA n** = **83**	**EA n** = **87**
*Distal points*		
LI4	95.2	89.7
EX2	72.3	75.9
LR3	68.7	67.8
GV20	50.6	43.7
SP6	33.7	36.8
LU7	24.1	24.1
PC6	3.6	5.7
*Local points*		
KI11	67.5	58.6
SP12	62.7	56.3
BL27	37.3	44.8
ST29	36.1	33.3
BL26	32.5	41.4
BL25	31.3	28.7
BL28	27.7	29.9
LR10	27.7	26.4
BL54	26.5	26.4
GB27	24.1	25.3
CV3	20.5	12.6
LR11	19.3	24.1
GB28	16.9	20.7
BL24	10.8	14.9
CV4	10.8	5.7
GB29	10.8	9.2
BL23	8.4	3.4
BL36	2.4	5.7
GB26	1.2	2.3
GB25	0.0	0.0

MA = Manual acupuncture, EA = Electro-acupuncture, GV = governor vessel channel, LI = large intestine channel, SP = spleen channel, LR = liver channel, PC = pericardium channel, EX = extra channel, LU = lung channel, BL = bladder channel, GB = gall bladder channel, KI = kidney channel, ST = stomach channel, CV = conception Vessel.

### Primary outcome

**Figure 2:**
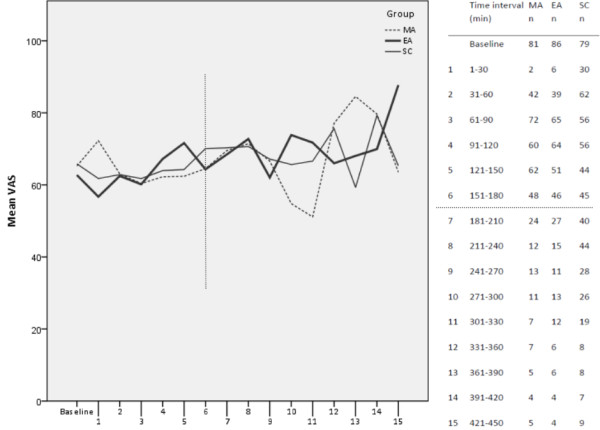
**Mean pain scores on a visual analogue scale (VAS) from time point 1 (baseline) to 15 (450 minutes).** MA = manual acupuncture, EA = electro-acupuncture, SC = standard care, n = number of valid observations at each time interval. After time interval 6, n < 41 in MA and EA. After time interval 8, n < 41 in SC. The model *with* interaction was used to identify time intervals when the three groups differed in pain scores on VAS.

Mean pain scores given using the VAS in relation to time are illustrated in Figure 2. Two different models were used to compare the outcomes of the three groups. The first model (without interaction) shows that mean estimated pain increased as labour progressed in all three groups with no differences between the groups (Table 3). The second model (with interaction) confirmed that there was an interaction between time and treatment (*p* = 0.03) and differences between the study groups in pain estimations occurred at the following time points: 120 minutes (MA pain scores lower than EA), 270 minutes (MA pain scores lower than EA), and 360 minutes (SC pain score lower than MA) (Figure 2). There were no differences in primary outcome measurements when analyses were performed according to the principles of ITT and PP respectively. All results presented in this article are analysed according to ITT.

**Table 3:** Estimated pain on VAS, and background factors associated with experienced pain

**Factor**	**Category**	**Valid observations N**	**Unadjusted Mean Estimates (SE)**	**Mean difference**^**1 **^**(95% CI for difference)**	***p***^***2***^	**Adjusted**^**3 **^**Mean Estimates (SE)**	**Mean difference**^**1 **^**(95% CI for difference)**	***p***^***2***^
**Treatment**					0.51			0.45
	SC (Ref)	561	67.6 (1.5)	Ref		69.0 (1.8)	Ref	
	MA	455	65.2 (1.5)	2.4 (-1.7-6.6)	0.25	66.4 (2.0)	2.6 (-1.7-6.9)	0.23
	EA	455	66.4 (1.5)	1.2 (-2.9-5.3)	0.57	68.5 (2.0)	0.6 (-3.6-4.8)	0.79
**Age**					0.03			0.04
	≤25 (Ref)	555	69.2 (1.4)	Ref		72.0 (1.8)	Ref	
	26-34	819	65.1 (1.2)	4.1 (0.6-7.7)	0.02	67.9 (1.4)	4.1 (0.6-7.7)	0.04
	35+	97	61.9 (3.3)	7.3 (0.3-14.3)	0.04	64.0 (3.3)	7.3 (0.3-14.3)	0.01
					0.21			0.82
**Higher education**	No (Ref)	796	67.3 (1.2)	Ref		68.2 (1.7)	Ref	
	Yes	661	65.1 (1.3)	2.2 (-1.3-5.6)	0.21	67.7 (1.9)	2.5 (-1.3-5.6)	0.82
**Time baseline (min)**					0.00			0.00
	0-30 (Ref)	246	64.5 (1.3)	Ref		63.1 (1.6)	Ref	
	31-60	38	58.6 (2.4)	5.9 (1.4-10.4)	0.01	57.1 (2.6)	6.0 (1.5-10.5)	0.01
	61-90	143	60.9 (1.6)	3.7 (0.5-6.8)	0.02	59.4 (1.8)	3.7 (0.5-6.9)	0.02
	91-120	193	62.5 (1.4)	2.0 (-1.2-5.3)	0.22	61.1 (1.7)	2.1 (-1.2-5.3)	0.22
	121-150	180	66.1 (1.5)	-1.6 (-5.1-1.9)	0.37	64.8 (1.7)	-1.7 (-5.2-1.8)	0.35
	151-180	157	68.0 (1.6)	-3.5 (-7.3-0.3)	0.07	66.7 (1.8)	-3.6 (-7.4-0.2)	0.06
	181-210	139	68.0 (1.6)	-3.4 (-7.4-0.6)	0.09	66.6 (1.9)	-3.5 (-7.5-0.5)	0.09
	211-240	91	70.6 (1.9)	-6.0 (-10.6 - -1.6)	0.01	69.2 (2.1)	-6.1 (-10.6 - -1.6)	0.01
	241-270	71	72.8 (2.2)	-8.2 (-13.2 - -3.3)	0.00	71.4 (2.4)	-8.2 (-13.2 - -3.3)	0.00
	271-300	52	71.0 (2.5)	-6.5 (-12.0 - -0.9)	0.02	69.6 (2.7)	-6.5 (-12.0 - -1.0)	0.02
	301-330	50	70.6 (2.6)	-6.1 (-11.9 - -0.3)	0.04	69.2 (2.8)	-6.1 (-12.0 - -0.3)	0.04
	331-360	38	72.2 (3.0)	-7.7 (-14.1 - -1.3)	0.02	70.9 (3.1)	-7.8 (-14.2 - -1.4)	0.02
	361-390	21	78.6 (3.8)	-14.1 (-22.0- -6.2)	0.00	77.4 (3.9)	-14.3 (-22.2 - -6.4)	0.00
	391-420	19	70.0 (4.1)	-5.4 (-13.9-3.0)	0.21	68.8 (4.2)	-5.6 (-14.1-2.8)	0.19
	421-450	15	84.7 (4.6)	-20.2 (-29.6 - -10.7)	0.00	83.8 (4.7)	-20.7 (-30.2 - -11.2)	0.00
	451-480	18	69.5 (4.5)	-5.0 (-14.3-4.3)	0.29	68.2 (4.6)	-5.0 (-14.4-4.3)	0.29

The main result is presented as the estimated VAS score for pain and corresponding standard error. VAS = Visual analogue scale. SC = Standard care. MA = Manual acupuncture. EA = Electro-acupuncture. Ref = reference category. ^1^Reference category-other category^. 2^Left-centered p-values represent overall comparisons between the levels of the factors while right-centred represent pairwise comparisons between each level with the reference level. ^3^Adjusted for: treatment group, age, education, and time from baseline, with no interactions.

### Secondary outcomes

Women’s assessment of relaxation did not differ between the groups. The unadjusted mean estimates were: MA 58.9 (SE 1.8), EA 60.0 (SE 1.7), and SC 62.8 (SE 1.7); *p* = 0.25, and the adjusted: MA 61.0 (SE 2.3), EA 62.9 (SE 2.3), and SC 64.6 (SE 2.1); *p* = 0.37.

The use of epidural analgesia was less frequent in the EA group than in the MA and SC groups (Table 4). Neither the mean time from baseline to epidural analgesia was administered (minutes), or the mean cervix dilatation (cm) at that point, differed between the groups (minutes: SC 335 (SE 29.7), MA 400 (SE 39.8), EA 330 (SE 36.0); *p* = 0.48, cm SC 5.6 (SD 1.6) MA 5.3 (SD 1.6) EA 5.3 (SD 1.1); *p* = 0.5). Fewer women in the two acupuncture groups were treated with TENS than in the SC group (Table 4). After the birth, most women reported that they had experienced worse pain than expected regardless of treatment although the majority said they had received sufficient pain relief (Table 4). Fewer women in the MA group reported that acupuncture was effective in reducing pain compared to the EA-group (Table 3). Only 55.3% (n = 42) women in the SC group were satisfied with the group allocation compared with 84.4% (n = 65) in the MA group and 88.8% (n = 71) in the EA group.

**Table 4:** Obstetric pain relief and postpartum assessments of the experience of labour pain (%)

	**MA**	**EA**	**SC**	**MA vs. SC**^**1**^	**EA vs. SC**^**1**^	**EA vs. MA**^**1**^
	**n** = **83**	**n** = **87**	**n** = **83**	***OR (CI)***^***2***^	***OR (CI)***^***2***^	***OR (CI)***^***3***^
*Pain relief*						
Nitrous oxide	95.1	95.4	93.8	1.89 (0.43-8.37)	1.52 (0.39-5.96)	0.80 (0.17-3.75)
Sterile water injections	12.2	4.7	10.0	1.15 (0.42-3.14)	0.40 (0.11-1.40)	0.35 (0.10-1.17)
TENS	14.5	12.6	48.1	0.17 (0.77-0.37)	0.16 (0.73-0.34)	0.94 (0.38-2.33)
Morphine	9.6	1.1	4.8	2.3 (0.64-8.0)	0.23 (0.25-2.1)	0.10 (0.01-0.85)
Epidural analgesia	61.4	46.0	69.9	0.62 (0.32-1.20)	0.35 (0.19-0.67)	0.57 (0.31-1.06)
*Women’s assessments postpartum*						
Pain worse than expected^4^	51.9	54.4	65.8	0.54 (0.27-1.05)	0.63 (0.33-1.23)	1.19 (0.62-2.26)
Sufficient pain relief^4^	76.6	81.0	73.7	1.31 (0.61-2.84)	1.68 (0.77-3.68)	1.28 (0.58-2.84)
Will choose the same treatment again^4^	60.9	68.0				1.14 (0.55-2.36)
Acupuncture effective for reducing pain^5^	44.4	67.1				2.44 (1.23-4.82)
Acupuncture effective for relaxation^5^	58.6	72.7				1.72 (0.84-3.52)

^1^Analysed by appropriate method (see Methods) and adjusted for age and education. SC = Standard Care MA = manual acupuncture EA = Electro-acupuncture OR = Odds Ratio CI = 95% Confidence interval SD = Standard Deviation TENS = Transcutaneous Electrical Nerve Stimulation ^2^SC is reference ^3^MA is reference ^4^Day after partus ^5^Immediately after partus.

Table 5 shows that obstetric and neonatal outcomes were very similar between the three study groups with the exception of duration of labour and blood loss. Women in the EA group had shorter labours than the SC group (Hazard ratio 1.44; 95% CI 1.06-1.97), and estimated blood loss was lower in the EA group than in the SC group with its slightly higher rate of caesarean section.

**Table 5:** Obstetric and neonatal outcomes

	**MA**	**EA**	**SC**	**MA vs. SC**^**1**^	**EA vs. SC**^**1**^	**EA vs. MA**^**1**^
	**n** = **83**	**n** = **87**	**n** = **83**			
**Obstetric outcomes**						
Mode of delivery (%)				*OR (CI)*^*2*^	*OR (CI)*^*2*^	*OR (CI)*^*3*^
Normal vaginal	74.7	74.7	74.7	0.97 (0.46-2.02)	0.94 (0.46-1.91)	0.97 (0.48-1.99)
Instrumental vaginal	16.9	19.5	12.0	1.52 (0.61-3.81)	1.93 (0.81-4.63)	1.27 (0.56-2.87)
Caesarean	8.4	5.7	13.3	0.64 (0.23-1.79)	0.41 (0.14-1.26)	0.65 (0.20-2.14)
Estimated blood loss >1000 ml (%)	4.8	2.3	12.2	0.36 (0.10-1.23)	0.14 (0.30-0.69)	0.40 (0.70-2.27)
Augmentation of labour (%)	63.9	54.7	60.5	1.19 (0.62-2.3)	0.81 (0.43-1.51)	0.68 (0.36-1.28)
Perineal trauma, third and fourth degree (%)	5.3	4.9	5.6	1.19 (0.28-5.16)	0.92 (0.21-3.92)	0.77 (0.18-3.29)
				*HR (CI*^*2*^*)*	*HR (CI)*^*2*^	*HR (CI)*
Duration of labour (minutes) mean(SD)	619 (378)	500 (319)	615 (398)	1.03 (0.75-1.41)	1.44 (1.06-1.97)	1.41 (1.03-1.91)
**Infant**				*OR (CI)*^*2*^	*OR(CI)*^*2*^	*OR (CI)*^*3*^
Transferred to neonatal clinic (%)	3.6	11.5	4.9	0.91 (0.19-4.31)	2.82 (0.82-9.68)	3.11 (0.81-11.98)
				*p*	*p*	*p*
Apgar score less than 7 at 5 min (%)	1.2	2.3	0	1.00	0.68	0.69
Cord arterial pH mean (SD)	7.3 (0.7)	7.2 (0.7)	7.3 (0.8)	1.00	0.52	0.45
Cord venous pH mean (SD)	7.3 (0.7)	7.3 (0.8)	7.3 (0.6)	1.00	0.68	0.69

^1^Analysed by appropriate method (see Methods) and adjusted for age and education. SC = Standard Care MA = manual acupuncture EA = Electro-acupuncture OR = Odds Ratio CI = 95% Confidence interval SD = Standard Deviation HR = Hazard Ratio ^2^SC is reference ^3^MA is reference.

Negative side effects were reported by 10% (n = 7) of the women in the MA-group and 7% (n = 5) of the women in the EA-group. These were mostly related to pain associated with the insertion of the needles, numbness and tiredness. The midwives reported adverse side effects in 2.8% (n = 2) of both MA and EA groups, and these were related to nausea. A majority of the women reported that they felt confident regarding the midwives’ skills when providing acupuncture treatment: MA 92.4% (n = 73), EA 88.8% (n = 71). In all three groups, the women had an overall positive experience of the midwife: MA 100% (n = 75), EA 97.5% (n = 78), and SC 98.7% (n = 74). In addition, a majority of the women in all three groups stated that they to a high extent received support from the midwife during labour MA 77.2% (n = 61), EA 83.5% (n = 66), and SC 80% (n = 60).

## Discussion

This trial was conducted with the aim of investigating the effect of acupuncture with two different stimulation protocols on women’s assessment of labour pain. The study demonstrated that acupuncture with combined manual and electrical stimulation was not more effective than acupuncture with only manual stimulation, or standard care without any form of acupuncture. Neither did we find any effect of the two acupuncture techniques on women’s assessment of relaxation during labour.

However, it was seen that women who received acupuncture with electrical and manual stimulation used less additional pain relief, including epidural analgesia, and had shorter labours than women in the standard care group. Women in the EA group were also more satisfied with the pain relief than women who received MA. The lower rate of epidural analgesia in the EA group could imply that the effect of acupuncture with electrical stimulation was underestimated in the study. This could be partially because women in the MA and SC groups had a higher rate of pharmacological pain relief and partially because women in the EA group remained longer in the study, which was discontinued at onset of epidural analgesia. These women therefore contributed with pain assessments in a later and more painful stage of labour than the other two groups. The ideal design of the study would have been to exclude the possibility of having any other form of pain relief than the acupuncture given in the two acupuncture groups, but this would not have been ethically justified. However, the option of having forms of pain relief with similar relieving mechanisms as acupuncture, namely TENS and sterile water injections could have been eliminated. For this reason, it is recommended that future studies avoid high intensity sensory treatment in control groups.

In this study, decisions about pain relief other than acupuncture were left to the women in labour and the midwives, and although we defined the study as an efficacy trial in the protocol [12] a more appropriate definition would be a pragmatic effectiveness trial.

We cannot, however, dismiss the possibility that acupuncture administrated in our study was not sufficiently intense, as only a small percentage of the women received a second treatment. The protocol stated that after the first 40 minutes of treatment, a second one should be administered after two hours; to increase the intensity of the treatment while minimising the risk of unnecessary immobilisation. However, the women were able move around more freely than we had anticipated and it could be discussed whether a *prolonged* first treatment would have been preferable but this needs to be evaluated in future studies. The midwives’ lack of time for placing the needles and connecting the EA device was given as a reason to that few women actually received a second treatment.

Physiological research suggests that acupuncture using a combination of manual and electrical stimulation could induce stronger pain relief than acupuncture with manual stimulation alone [11]. During labour, the pain system is highly sensitised, which places high demands on the given pain relief. Postoperative pain is in some ways similar to labour pain with a large acute inflammatory component, and the effect of acupuncture on this type of pain has been questioned [18]. This is in contrast to chronic pain conditions, for which acupuncture has been shown to be superior to sham treatments [19].

The finding that acupuncture had no significant effect on pain, yet reduced the frequency of use of other forms of pain relief has been observed in previous studies [5,7]. The reduction of use of pain relief must however be interpreted with some caution since this may be influenced not only by the woman in labour but also by the care provider and local culture within the labour ward [20]. However, our finding that epidural analgesia in the EA group was reduced in both the settings used, located in different parts of Sweden, supports the interpretation that it was the women’s choice, suggesting that electro-acupuncture had a pain relieving effect that made epidural analgesia unnecessary. The use of epidural analgesia in the SC group was higher than expected, 70% versus 54% of the 4029 eligible women, and this finding could possibly be related to the fact that women were not recruited to the study if being too close to partus, or the higher rate of dissatisfaction with the allocation. Women in the EA group spent less time in labour than women in the SC group and this is likely a result of the lower use of epidural analgesia in the EA group. When we excluded women receiving epidural analgesia there were no differences in time spent in labour between the groups (not shown), which is consistent with results from a previous study [4].

A recent review concluded that acupuncture may have a role in reducing labour pain, however more research is needed [21]. The authors addressed some of the methodological problems with acupuncture research, and in an attempt to make our study as well described as possible a full description of the study design has been published previously [12].

Ideally, an RCT should be blinded, but there is a problem with placebo controls in acupuncture research, since they possibly have similar physiological effects as acupuncture in the activation of the endogenous opioid system [22,23]. We therefore decided not to use a placebo control group and hence blinding was impossible. There is a risk that both midwives and the women could be biased in favour for the acupuncture treatment since none of them were blinded. However, this concern would apply to both types of acupuncture: MA and EA. The difference in pain scores seen when comparing EA with SC but not between MA and SC indicates that there was an actual difference and not only by preference of the women and midwives.

The VAS is the most commonly used instrument for assessment of pain and has been validated to detect changes in pain intensity [24,25]. It has also been used in nearly all acupuncture studies for labour pain [3-6,8,9]. The rationale for choosing the VAS was the feasibility and intelligibility of the instrument during intense situations such as labour, and that no superior instrument is available.

The midwives’ skills in acupuncture treatment may possibly have affected the outcomes, and a full description of their training and experience has been published previously [12]. The length of the training may have been too short to gain sufficient skills to optimize the treatment [26]. However previous studies have demonstrated that the number of hours of training does not affect the results of treatment [27]. In addition, a relative small number of acupuncture points were used in this study, which makes the risk of inadequate training affecting the results smaller. The relatively high number of midwives (n = 38) who participated in this study compared with, for instance, the study by Skilnand [4] and colleagues (n = 6) could also be important. They may not have had the opportunity to gain sufficient skills since the number of treatments given by each midwife was low. To assure that the intervention procedures were performed correctly, the course included theoretical and practical sessions of MA and EA, which were repeated every semester. In addition, all midwives had access to a website which included instructional videos and written information about the study. Intermittent check-ups at the delivery wards were made by the first author (LV) to assure that the interventions followed study protocol.

## Conclusion

Our findings suggest that acupuncture does not reduce women’s experience of labour pain, neither with manual stimulation nor with combined manual and electrical stimulation. However, it was found that the EA group had a lower frequency of epidural analgesia thus indicating that the effect of acupuncture with electrical stimulation may have been underestimated. These findings were obtained in a context with free access to other forms of pain relief, including epidural analgesia.

## Competing interest

All authors have completed a declaration of competing interests and declare no financial relationships that may be relevant to the submitted work; and have no non-financial interests that may be relevant to the submitted work.

## Authors’ contributions

LV, ES, ES-V, UW and LBM participated in the study design. ES, UW and LBM obtained funding for the study. LV collected the data. LV was trial manager. LV analyzed the data and HP provided statistical expertise. LV drafted the article, which was then revised for important intellectual content by all authors. All authors read and approved the final manuscript.

## Pre-publication history

The pre-publication history for this paper can be accessed here:

http://www.biomedcentral.com/1472-6882/14/187/prepub

## References

[B1] Mårtensson L Kvist LJ Hermansson E A national survey of how acupuncture is currently used in midwifery care at Swedish maternity units Midwifery 2011 27 1 87 92 10.1016/j.midw.2009.11.005 20092915

[B2] Cho SH Lee H Ernst E Acupuncture for pain relief in labour: a systematic review and meta-analysis Br J Obstet Gynaecol 2010 117 8 907 920 10.1111/j.1471-0528.2010.02570.x 20438555

[B3] Hantoushzadeh S Alhusseini N Lebaschi AH The effects of acupuncture during labour on nulliparous women: a randomised controlled trial Aust N Z J Obstet Gynaecol 2007 47 1 26 30 10.1111/j.1479-828X.2006.00674.x 17261096

[B4] Skilnand E Fossen D Heiberg E Acupuncture in the management of pain in labor Acta Obstet Gynecol Scand 2002 81 10 943 948 10.1034/j.1600-0412.2002.811008.x 12366485

[B5] Borup L Wurlitzer W Hedegaard M Kesmodel US Hvidman L Acupuncture as pain relief during delivery: a randomized controlled trial Birth 2009 36 1 5 12 10.1111/j.1523-536X.2008.00290.x 19278378

[B6] Ziaei S Hajipour L Effect of acupuncture on labor Int J Gynaecol Obstet 2006 92 1 71 72 10.1016/j.ijgo.2005.09.008 16253255

[B7] Ramnero A Hanson U Kihlgren M Acupuncture treatment during labour–a randomised controlled trial Br J Obstet Gynaecol 2002 109 6 637 644 12118641

[B8] Nesheim BI Kinge R Berg B Alfredsson B Allgot E Hove G Johnsen W Jorsett I Skei S Solberg S Acupuncture during labor can reduce the use of meperidine: a controlled clinical study Clin J Pain 2003 19 3 187 191 10.1097/00002508-200305000-00006 12792557

[B9] Mårtensson L Stener-Victorin E Wallin G Acupuncture versus subcutaneous injections of sterile water as treatment for labour pain Acta Obstet Gynecol Scand 2008 87 2 171 177 10.1080/00016340701797799 18231884

[B10] White A Cummings M Barlas P Cardini F Filshie J Foster NE Lundeberg T Stener-Victorin E Witt C Defining an adequate dose of acupuncture using a neurophysiological approach - a narrative review of the literature Acupunct Med 2008 26 2 111 120 10.1136/aim.26.2.111 18591910

[B11] Zhao Z-Q Neural mechanism underlying acupuncture analgesia Prog Neurobiol 2008 85 4 355 375 10.1016/j.pneurobio.2008.05.004 18582529

[B12] Vixner L Martensson LB Stener-Victorin E Schytt E Manual and electroacupuncture for labour pain: study design of a longitudinal randomized controlled trial Evid Based Complement Alternat Med 2012 Volume 2012 (2012), Article ID 943198 9 10.1155/2012/943198 PMC3345610 22577468

[B13] Moher D Hopewell S Schulz KF Montori V Gotzsche PC Devereaux PJ Elbourne D Egger M Altman DG CONSORT 2010 Explanation and Elaboration: updated guidelines for reporting parallel group randomised trials BMJ 2010 340 mar23 1 c869 c879 10.1136/bmj.c869 20332511 PMC2844943

[B14] MacPherson H Altman DG Hammerschlag R Youping L Taixiang W White A Moher D Revised standards for reporting interventions in clinical trials of acupuncture (STRICTA): extending the CONSORT statement PLoS Med 2010 7 6 e1000261 10.1371/journal.pmed.1000261 20543992 PMC2882429

[B15] Pyne D Shenker NG Demystifying acupuncture Rheumatology 2008 47 8 1132 1136 10.1093/rheumatology/ken161 18460551

[B16] Wang SM Kain ZN White P Acupuncture analgesia: I. The scientific basis Anesth Analg 2008 106 2 602 610 10.1213/01.ane.0000277493.42335.7b 18227322

[B17] Todd KH Funk KG Funk JP Bonacci R Clinical significance of reported changes in pain severity Ann Emerg Med 1996 27 4 485 489 10.1016/S0196-0644(96)70238-X 8604867

[B18] Meissner W The role of acupuncture and transcutaneous-electrical nerve stimulation for postoperative pain control Curr Opin Anaesthesiol 2009 22 5 623 626 10.1097/ACO.0b013e32832fbdf1 19593119

[B19] Vickers AJ Cronin AM Maschino AC Lewith G MacPherson H Foster NE Sherman KJ Witt CM Linde K Acupuncture T Acupuncture for chronic pain individual patient data meta-analysis Arch Intern Med 2012 172 19 1444 1453 10.1001/archinternmed.2012.3654 22965186 PMC3658605

[B20] Schytt E Waldenstrom U Epidural analgesia for labor pain: whose choice? Acta Obstet Gynecol Scand 2010 89 2 238 242 10.3109/00016340903280974 19824867

[B21] Smith CA Collins CT Crowther CA Levett KM Acupuncture or acupressure for pain management in labour Cochrane Database Syst Rev 2011 Issue 7 Art. No.: CD009232 doi:10.1002/14651858.CD009232 10.1002/14651858.CD009232 21735441

[B22] Birch S A review and analysis of placebo treatments, placebo effects, and placebo controls in trials of medical procedures when sham is not inert J Altern Complement Med 2006 12 3 303 310 16646730 10.1089/acm.2006.12.303

[B23] Lundeberg T Lund I Naslund J Thomas M The Emperor’s sham - wrong assumption that sham needling is sham Acupunct Med 2008 26 4 239 242 10.1136/aim.26.4.239 19098696

[B24] Choiniere M Melzack R Girard N Rondeau J Paquin MJ Comparisons between patients’ and nurses’ assessment of pain and medication efficacy in severe burn injuries Pain 1990 40 2 143 152 10.1016/0304-3959(90)90065-L 2308761

[B25] Joyce CR Zutshi DW Hrubes V Mason RM Comparison of fixed interval and visual analogue scales for rating chronic pain Eur J Clin Pharmacol 1975 8 6 415 420 10.1007/BF00562315 1233242

[B26] WHO WHO Guidelines on Basic Training and Safety in Acupuncture 1999 [http://apps.who.int/medicinedocs/en/d/Jwhozip56e/]

[B27] Cummings M Modellvorhaben Akupunktur - a summary of the ART, ARC and GERAC trials Acupunct Med 2009 27 1 26 30 10.1136/aim.2008.000281 19369191

